# Quasi-Optical Terahertz Microfluidic Devices for Chemical Sensing and Imaging

**DOI:** 10.3390/mi7050075

**Published:** 2016-04-25

**Authors:** Lei Liu, Zhenguo Jiang, Syed Rahman, Md. Itrat Bin Shams, Benxin Jing, Akash Kannegulla, Li-Jing Cheng

**Affiliations:** 1 Department of Electrical Engineering, University of Notre Dame, 275 Fitzpatrick, Notre Dame, IN 46556, USA; zjiang@nd.edu (Z.J.); srahman@nd.edu (S.R.); mshams@nd.edu (M.I.B.S.); 2 Department of Chemical Engineering and Material Science, Wayne State University, 5050 Anthony Wayne Dr., Detroit, MI 48202, USA; Jingbenxin@gmail.com; 3 School of Electrical Engineering and Computer Science, Oregon State University, Corvallis, OR 97330, USA; kannegua@oregonstate.edu (A.K.); chengli@eecs.oregonstate.edu (L.-J.C.)

**Keywords:** terahertz, microfluidic, quasi-optical, frequency domain, chemical sensing and imaging, laminar flow, label free, molecule diffusion

## Abstract

We first review the development of a frequency domain quasi-optical terahertz (THz) chemical sensing and imaging platform consisting of a quartz-based microfluidic subsystem in our previous work. We then report the application of this platform to sensing and characterizing of several selected liquid chemical samples from 570–630 GHz. THz sensing of chemical mixtures including isopropylalcohol-water (IPA-H_2_O) mixtures and acetonitrile-water (ACN-H_2_O) mixtures have been successfully demonstrated and the results have shown completely different hydrogen bond dynamics detected in different mixture systems. In addition, the developed platform has been applied to study molecule diffusion at the interface between adjacent liquids in the multi-stream laminar flow inside the microfluidic subsystem. The reported THz microfluidic platform promises real-time and label-free chemical/biological sensing and imaging with extremely broad bandwidth, high spectral resolution, and high spatial resolution.

## 1. Introduction

Chemical and biochemical sensing has been increasingly more important in security, environmental, medical and clinical applications [1,2,3,4]. However, most current sensing and probing techniques are time-consuming, and require specific expertise and expensive equipment [5]. In addition, many of these techniques require chemical alteration of samples or labeling with fluorescent chromophores prior to detection and analysis [6]. For example, current DNA hybridization detection is mainly based on fluorescent labeling, which introduces unwanted preliminary processing steps and eventually modifies the DNA sample under test, resulting in system inefficiency and low accuracy [7]. Alternative label-free methods, such as mass sensitive [8], electrochemical [9], and acoustic wave [10], have been intensively studied, but no approach has been mature enough to provide performance as competitive as standard fluorescent-based systems.

With the emerging advances in device and circuit technologies in the terahertz (THz) regime (0.1 to 10 THz), electromagnetic waves in this frequency range have found many promising applications in noninvasive, label-free and remote sensing for many substances of interest (e.g., chemicals, explosives, drugs) owing to the strong interaction between THz waves and low-energy events (e.g., molecular rotation, torsion, vibration, as well as inter- and intra-molecular hydrogen-bonding) in chemical samples [11,12,13,14,15,16]. However, the THz sensing capability for liquid samples has been greatly limited due to strong undesired THz absorption introduced by hydrogen-bonding in the aqueous media or matrices (e.g., water) [17,18,19,20]. In view of this, researchers have started to combine THz technology with microfluidic devices to reduce the wave traveling path in liquid samples for leveraging THz absorption [21,22,23,24]. Although THz time-domain spectroscopy (THz-TDS) systems are widely employed for the above purposes, their spectral and spatial resolution for chemical sensing and imaging are generally inferior [13,25].

In this paper, we first review the development of a frequency-domain quasi-optical THz sensing and imaging platform consisting of a quartz-based microfluidic subsystem supporting four-stream laminar flow. We then applied the platform to sensing and imaging of a variety of chemicals, mixtures as well as molecular diffusion at liquid-liquid interface. The employed THz frequency-domain spectroscopy (THz-FDS) offers much improved spectral (better than ~10 kHz) and spatial resolution while the microfluidic device exhibits significant advantages including low analyte consumption, well-confined multi-stream flow, fast dynamics and autonomous operation [26], making the platform an extremely versatile system for high performance chemical/biological sensing and imaging. 

## 2. Experimental Section

### 2.1. Experimental Setup

**Figure 1:**
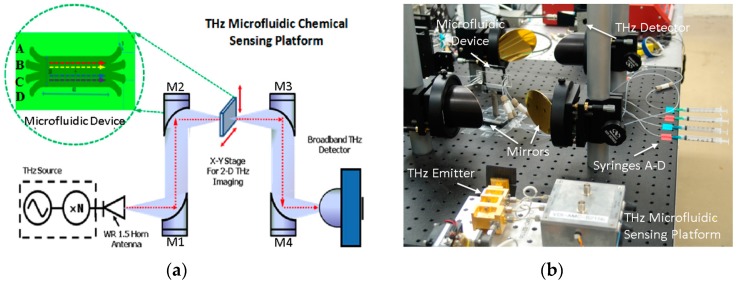
The THz microfluidic chemical sensing and imaging platform: (**a**) a schematic of the system comprising a quasi-optical THz-FDS spectroscopy and a four-channel microfluidic subsystem; (**b**) a photo showing the actual experimental setup. Liquid samples are delivered to the microfluidic chip through the four syringes A–D [26].

Figure 1a schematically illustrates the THz sensing and imaging platform we developed for this work. The quasi-optical THz-FDS system was reported in our previous work [26,27,28]. In this system, an amplifier multiplier chain (AMC, Virginia Diodes, Charlottesville, VA, USA) is employed as the THz emitter to provide THz radiation from 570 to 630 GHz with an average power level of ~1 mW. Much broader frequency coverage (e.g., 0.1–1.0 THz) can be achieved by using multiple THz emitters for different THz bands. The THz wave emitted from the frequency multiplier chain is first collimated and then focused onto the microfluidic device by the off-axis parabolic mirrors M1 and M2. The transmitted THz signal is then collimated and focused onto the quasi-optical THz detector for broadband and room-temperature operation [29]. In order to enable two-dimensional (2-D) THz mapping and imaging, the microfluidic device is mounted on a computer-controlled *X*-*Y*-*Z* positioning stage (not shown). As pointed out in our previous work, the spatial resolution of the THz microfluidic system was designed to be approximately 0.5 mm at the sampling position [26].

### 2.2. Quartz-Based Microfluidic Device

**Figure 2:**
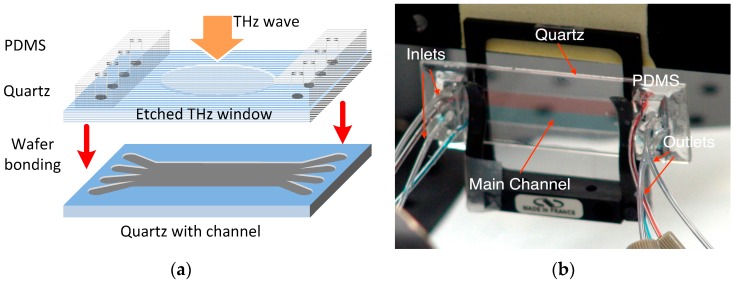
(**a**) The fabrication of the quartz-based microfluidic device using wafer bonding; (**b**) A two-stream laminar flow (with red and blue dyes at an injection rate of 100 μL/min) formed inside the device main channel demonstrating that the design and fabrication of the device were successful.

As seen in Figure 1b, the key device in this sensing platform is the four-channel (with multiple inlets/outlets) quartz-based microfluidic subsystem. The microfluidic subsystem was fabricated by bonding two fused quartz substrates (1 mm-thick, 1 in. × 3 in. in dimension) as shown in Figure 2a. Quartz substrates were chosen for building this microfluidic chip because of their low insertion loss (THz), low dielectric constant and optical transparency. The main channel of the device was etched to have a depth of 50 μm and a width of 1 cm, leading to an inner volume of ~20 μL. In order to support four-stream laminar flow, four through holes were drilled on the top quartz at both ends of the main channel. Two polydimethylsiloxane (PDMS) slabs were then bonded to the top slide with corresponding through holes aligned with those on the quartz substrate. Polytetrafluoroethylene (PTFE) tubings were then inserted to the through holes for delivery and drainage of the fluid, respectively. In order to reduce the “standing-wave” (or “cavity”) effect due to the relatively thick quartz substrates (*i.e.*, 2 mm thickness or 80 GHz in period), thinner and wedged THz window configuration can be micromachined on both the top and bottom quartz slides as shown in Figure 2a. During the chemical sensing and imaging experiments, four syringes (A–D) as seen in Figure 1b were used to inject liquid chemical samples into the main channel with one sample at a time or multiple samples simultaneously to form a multi-stream laminar flow (*i.e.*, 2–4 streams). As shown in Figure 2b, an initial test by injecting red and blue dyes simultaneously into the inlets A and D respectively at a rate of 100 μL/min clearly showed the formation of a stable two-stream laminar flow, demonstrating the success of the design and fabrication of the microfluidic subsystem. Finally, the operation of the entire system including frequency scan for THz spectroscopy and two-dimensional (2D) spatial scan for imaging were fully controlled and automated by a computer using a home-written LabView interface. 

### 2.3. Sensing Platform Characterization and Data Acquisition

**Figure 3:**
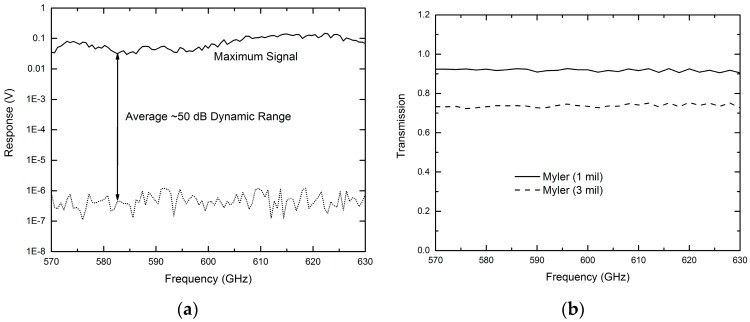
(**a**) Measured system dynamic range showing an average of 50 dB over the frequency range of 570–630 GHz; (**b**) measured transmission spectrum of Mylar thin films [27].

During the operation of the sensing platform, the THz emitter is modulated with an optical chopper at 1 kHz. The modulated DC voltage output from the quasi-optical THz detector is fed into a preamplifier (×100) and a lock-in amplifier for processing. Before applying this THz microfluidic platform to chemical sensing and imaging, the system’s performance was examined by measuring dynamic range and Mylar thin films. Figure 3a shows the measured maximum signal level and noise floor from 570 to 630 GHz. An average system dynamic range of ~50 dB has been demonstrated which is considered enough for many applications. Figure 3b shows the measurement results for a 1-mil thick Mylar thin film (solid line) and a 3-mil Mylar thin film (dashed line) [27]. Both curves were obtained by normalizing the measured data to that of background signal. The expected lower transmittance (~73%) for the 3-mil Mylar as compared to that of the 1-mil Mylar (~92%), as well as the flat linear frequency response observed from both samples clearly show the system’s capability for sensing different samples with a measurement accuracy better than 2% [27].

For a prototype demonstration, the developed frequency domain quasi-optical THz microfluidic platform has been applied to chemical sensing and imaging by performing frequency scan in the band of 570–630 GHz. The transmission spectra of background, the empty microfluidic device, as well as a variety of microfluidic-chip confined chemicals and their mixtures were acquired automatically by performing frequency scan at a resolution of 0.6 GHz with a speed of 10 ms per data point using the LabView interface described in Section 2.2.

## 3. Results and Discussion

### 3.1. THz Sensing of Chemicals

**Figure 4:**
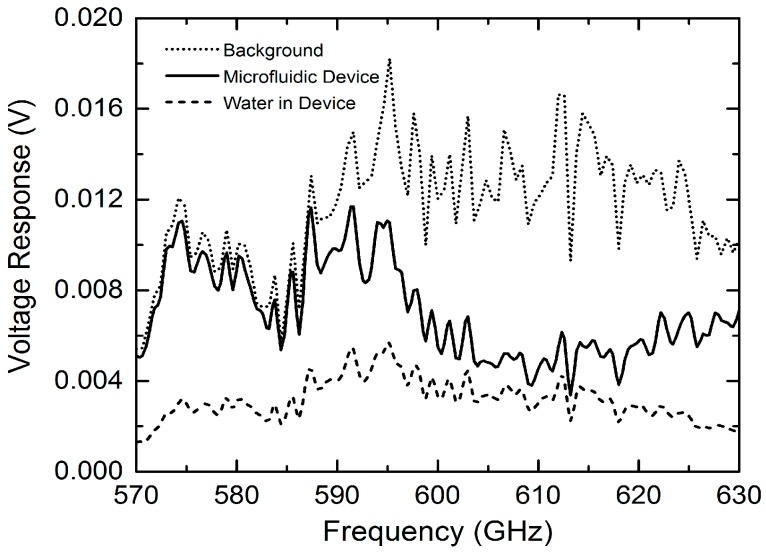
Measured THz responses (raw data without normalization) for background (ambient), empty microfluidic device and water-filled microfluidic device, respectively [26].

The measured THz transmission spectral without normalization for background, empty microfluidic device and deionized water filled device, respectively, are shown in Figure 4 [26]. The measured signal level (voltage response) for the empty microfluidic device is slightly lower as compared to that of the background in the region of 570–590 GHz, indicating the low-loss properties of the microfluidic device. However, due to the standing wave effect discussed in Section 2, a large transmission loss is observed from 590 to 630 GHz (*i.e.*, half period of ~40 GHz). This undesired standing wave effect can be effectively reduced by incorporating thin and wedged THz window on the quartz microfluidic device (see Figure 2a). The THz transmission signal level for the water-filled device was further reduced. However, the detected signal level falls well within the system’s dynamic range, demonstrating that the quartz microfluidic device with a 50-μm THz transmission path is a suitable design for sensing chemicals in a well-controlled aqueous environment.

**Figure 5:**
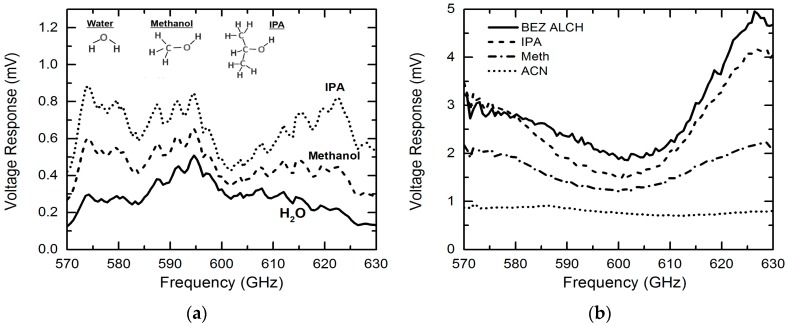
(**a**) Raw data of the THz spectra responses for isopropylalcohol (IPA), Methanol and water; (**b**) Comparison of normalized (to water) THz spectra responses for a variety of chemicals (Benzyl alcohol (BEZ ALCH), isopropylalcohol (IPA), methanol (Meth) and acetonitrile (ACN)), demonstrating the system’s capability for discriminating different chemicals.

In our previous work, we focused on sensing and imaging of only isopropylalcohol (IPA), water and their mixtures [26]. In this paper, we extend our research by applying the sensing platform to characterize a variety of chemicals. In Figure 5a, we first compare the measured THz transmission responses (raw data without normalization) of IPA, methanol and water. The THz absorption increases in the following order: IPA < methanol < water, which is consistent with the findings from previous research [25]. This phenomenon can be explained using the different hydrogen bond densities in these three liquids. As discussed in Reference [26], the hydrogen bond densities of water, methanol, and isopropanol are calculated to be 1.2 × 10^23^, 2.7 × 10^22^ and 1.4 × 10^22^ cm^−3^, respectively [30,31,32,33]. Different from infrared spectroscopy, THz waves interact significantly with hydrogen bonds—liquids with higher hydrogen bond density tend to yield high THz absorption. This explains the different THz transmission levels observed in the three liquid samples in Figure 5a. To better compare the sensing results for different chemicals, we normalized the transmission response raw data for each chemical to that of water. Figure 5b shows the normalized THz transmission features for Benzyl (BEZ) alcohol, IPA, methanol and acetonitrile (ACN) in the same frequency band, *i.e.*, 570–630 GHz. A response peak is observed at ~625 GHz for all chemicals and this is believed to be introduced by the well-know standing-wave effect [26]. Different from BEZ alcohol, IPA and methanol that have a lower THz absorption due to lower hydrogen bond densities, pure ACN shows larger THz absorption than water due to the relatively strong resonance between THz waves and the vibration mode of individual ACN cluster. 

### 3.2. THz Sensing of Chemical Mixtures

**Figure 6:**
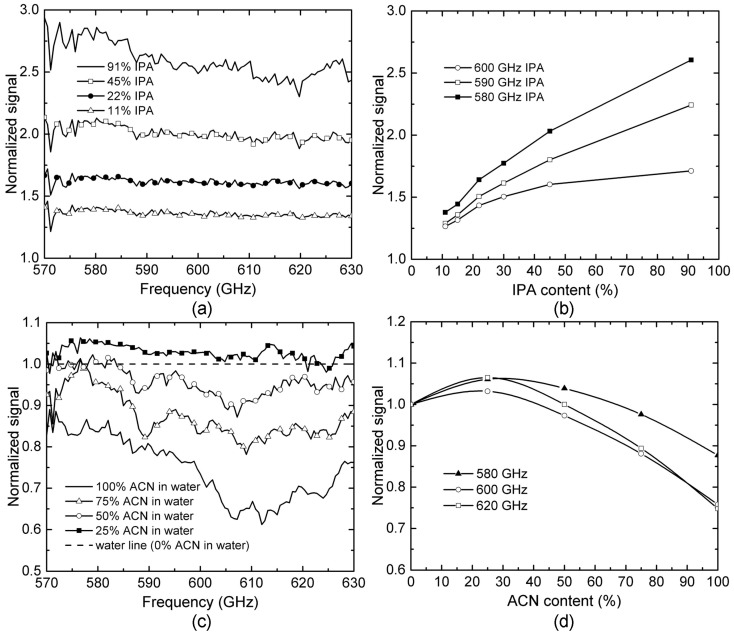
THz microfluidic sensing and analysis of IPA-H_2_O and ACN-H_2_O mixtures: (**a**) normalized (to water) THz transmission spectra of the IPA-H_2_O mixtures with IPA concentration ranging from 10% to 91%; (**b**) output THz signal responses at three selected frequencies showing strong linear relationship of the signal as a function of IPA concentration; (**c**) normalized (to water) THz transmission spectra of the ACN-H_2_O mixtures with different ACN concentrations; (**d**) output THz signal responses at three selected frequencies functions of IPA concentration. Nonlinear relationships observed showing completely different hydrogen dynamics and THz absorption mechanisms (as compared to IPA-H_2_O mixtures).

In order to reveal the details of the above observation, we further studied and compared the THz transmission spectra for IPA-H_2_O and ACN-H_2_O mixtures with various concentrations. In this study, all spectra data were processed by normalizing to water, and the known cavity effect was removed numerically. As seen in Figure 6a, the THz signal intensity increases when the IPA concentration is increased because the decreasing in hydrogen bond density leading to lower THz absorption based on what was observed in Figure 5. The transmission signals for IPA-H_2_O mixtures at three different frequencies, *i.e.*, 580, 590 and 600 GHz, were plotted as functions of the IPA concentration as shown in Figure 6b. Although slight nonlinearity is found, all these three plots show monotonous relationship between THz response and IPA concentration.

Similar experiments have been also performed to characterize ACN-H_2_O mixtures, as shown in Figure 6c, with ACN concentrations ranging from 0% to 100%. Although pure AN has higher THz absorption than water (*i.e.*, lower normalized signal intensity is expected), surprisingly, the normalized signal intensity increases first for smaller ACN concentration (<25%) and then decreases for larger concentrations. Figure 6d shows the normalized THz transmission response as a function of ACN concentration at three selected frequencies, *i.e.*, 580, 600 and 620 GHz. Different from the monotonous relation that has been observed for IPA-H_2_O mixtures (see Figure 6b), THz responses for ACN-H_2_O mixtures show a strong nonlinear and non-monotonous relation to ACN concentration, with the lowest THz absorption (maximum signal intensity) occurring for an ACN concentration around 25%. This observation reveals completely different hydrogen bond dynamics and THz absorption mechanisms in IPA-H_2_O and ACN-H_2_O mixtures.

Similar nonlinearity for ACN-H_2_O mixtures has been reported in studies using infrared spectroscopy [34,35]. The absorption of THz energy by liquid samples can be attributed to the resonance between THz waves and vibration modes of molecular clusters formed by either a hydrogen bond or dispersion force. From Figure 5, we can see that higher hydrogen bond density leads to more molecular clusters with the resonating vibration mode. However, when ACN is mixed with water, the situation is different. In the low ACN concentration regime, each ACN molecule only forms one hydrogen bond with water and thus behaves as an end-cap agent that suppresses hydrogen bond number/density between water molecules. The increase of ACN in a mixture could lead to the decreasing concentration of water cluster resulting in lower THz absorption [34]. However, once the fraction of ACN in water is high enough (>25%), ACN clusters will be formed through dipole-dipole interaction. The vibration of the individual ACN cluster leads to higher THz absorption. As a result, in the high ACN concentration regime, THz absorption increases with ACN concentration, as seen in Figure 6d [36]. 

### 3.3. THz Imaging of Molecular Diffusion

**Figure 7:**
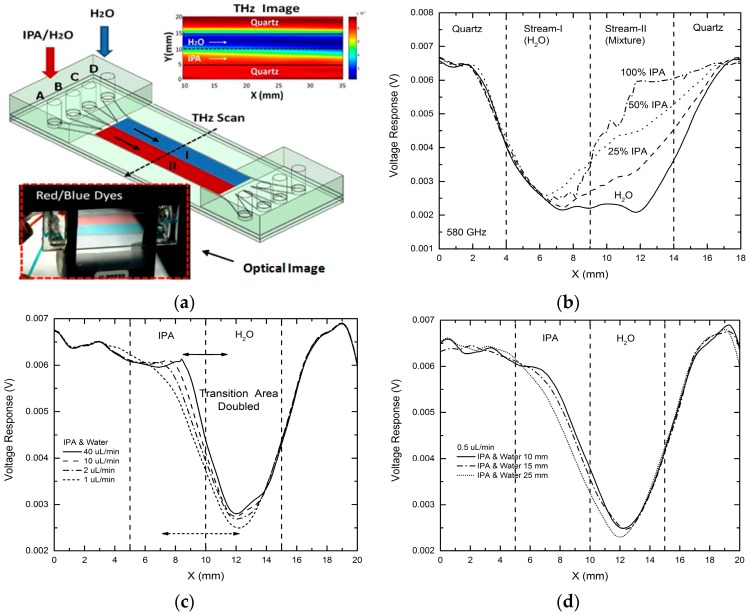
THz chemical sensing and imaging (580 GHz) for studying molecular diffusion at liquid-liquid interfaces of two-stream laminar flows: (**a**) schematic showing the THz imaging of two-stream laminar flow inside the device (lower left inset shows an optical image of a two-stream laminar flow with red and blue dyes at an injection rate of 100 μL/min; upper right inset shows a THz 2-D scanning image of a laminar flow formed by water and IPA; (**b**) 1-D THz scanning results for two-stream laminar flows formed by water and IPA-H_2_O mixtures [26]; (**c**) 1-D THz scanning across the device at *X* = 30 mm for laminar flows formed by water and IPA at different injection rate from 40 to 0.5 μL/min. The transition region at the interface is nearly doubled; (**d**) 1-D THz scanning at different positions of the device (*X* = 10–25 mm) for a laminar flow by water and IPA at a rate of 0.5 μL/min. The transition at the liquid-liquid interface changes from 5.8 to 6.2 mm when *X* changes from 10 to 25 mm, showing stronger diffusion at the outlets side of the microfluidic device.

In order to apply this THz microfluidic platform to study molecular diffusion and potential chemical reactions between two liquid chemical samples, we attempted THz mapping for a two-stream laminar flow situation, initially tested by using red and blue dyes (see Figure 7a, lower inset). For the above purpose, water and IPA-H_2_O mixtures having variant IPA concentrations were injected into the microfluidic device at an injection rate of 100 μL/min to form a two-stream laminar flow. THz image with 20 × 200 (*X* × *Y*) pixels was taken at 580 GHz by performing 2-D scanning of the device [26]. As shown in Figure 7a (upper inset), the interface between the two streams was clearly seen. Figure 7b shows the measurement results of one-dimensional (1-D) THz scanning across the microfluidic device (*Y*-direction) at *X* = 30 mm. As expected, the distinguishable THz transmission levels were detected across the entire device region in response to different chemical streams (Stream-I (H_2_O) region and Stream-II (mixture) region). It is clearly observed that the signal level in the Stream-II region changed as expected when the IPA concentration for Stream-II region was increased from 0% to 100%, demonstrating that the approach of using multi-stream microfluidic device indeed enables THz sensing of molecular diffusion and potential chemical reactions at the interfaces between adjacent liquid samples [26]. 

For a prototype demonstration of this imaging capability to study molecular diffusion, we performed 1-D THz scan at the same position (*i.e.*, across the channel at *X* = 30 mm) for laminar flows formed by water and IPA, for different injection rates from 40 to 1 μL/min as shown in Figure 7c. With decreasing of the flow speed, the transition width at the liquid-liquid interface was observed to increase from ~3 to ~6 mm. The transition area almost doubled at a lower flow speed, indicating higher diffusion level between the two liquids was detected. We then kept the injection rate at 0.5 μL/min and performed THz 1-D scan across the microfluidic device at different positions from *X* = 10 mm to *X* = 35 mm. As seen in Figure 7d, the transition width at the interface changes from 5.8 mm (at *X* = 10 mm) to 6.2 mm (at *X* = 35 mm), also showing stronger diffusion at the outlets side of the device than that at the inlets side of the device, as expected. This same approach can be adopted to study and visualize chemical reactions between two or more chemicals at their laminar flow interfaces.

## 4. Conclusions

A frequency domain quasi-optical THz chemical sensing and imaging platform consisting of a quartz-based microfluidic device supporting multi-stream laminar flow has been developed. The performance of this sensing platform has been fully characterized. This system has been successfully applied to sensing several selected liquid chemical samples from 570 to 630 GHz. THz spectroscopic sensing of chemical mixtures including IPA-H_2_O and AN-H_2_O mixtures with different concentrations have been successfully demonstrated, revealing different hydrogen dynamics and absorption mechanisms in different mixture systems. 2-D mapping and imaging of two-stream laminar flows as well as molecule diffusion at the liquid-liquid interface has been performed and discussed. The reported THz microfluidic platform promises real-time and label-free chemical/biological sensing and imaging with extremely broad bandwidth, high spectral resolution, and high spatial resolution.
